# The effectiveness of physiotherapy for chronic headaches in patients with temporomandibular disorders: a systematic review

**DOI:** 10.3389/fresc.2025.1647927

**Published:** 2025-09-23

**Authors:** Charlène Quilghini, Julian Lefflot, Kim Buchholtz

**Affiliations:** ^1^ Department of Health, LUNEX S.A., Differdange, Luxembourg; ^2^ Luxembourg Health & Sport Sciences Research Institute A.s.b.l., Differdange, Luxembourg

**Keywords:** systematic review, conservative care, temporomandibular pain, orofacial pain, headaches

## Abstract

**Background:**

Chronic headaches (CH) affect approximately 1 billion people globally, with women having three to five times higher prevalence. The estimated cost in Europe is €173 billion. Recent studies suggest a strong link between chronic headaches and temporomandibular disorders (TMD), which are characterized by orofacial pain, temporomandibular joint symptoms, and limited mandibular movement. Physiotherapy for these disorders often involves addressing muscle spasms through massage, trigger point therapy, and active stretching.

**Objective:**

This systematic review aimed to assess the effectiveness of temporomandibular joint (TMJ) physiotherapy for patients with chronic headaches (CH) and temporomandibular disorders (TMD).

**Methods:**

A systematic literature search was performed in January 2025 using the PICOS framework and relevant MeSH terms across the PubMed, PEDro, and Cochrane databases. Two reviewers independently screened studies, with a third reviewer resolving disagreements. Five randomized controlled trials (RCTs) met the inclusion criteria. Data extraction and study characteristics were analyzed, and the risk of bias was assessed using the Cochrane RoB2 tool.

**Results:**

The review identified five studies, suggesting that physiotherapy may benefit these patients. Three studies showed significant improvements in headache intensity and frequency following TMJ or orofacial physiotherapy. One study favored the control group, and one showed no significant difference. However, variability in study quality, therapist roles, and poorly reported interventions limited comparability and prevented meta-analysis. The findings point to potential benefits of physiotherapy for managing chronic headaches and TMD but underscore the need for more standardized research.

**Conclusion:**

This review highlights the potential of multidisciplinary treatments for patients with chronic headaches and temporomandibular disorders. However, due to the variability in treatment protocols and outcome measures, further research is needed to confirm these findings and standardize protocols for more reliable and consistent results.

## 1 Introduction

Chronic headaches (CH) are common conditions affecting 1 billion of the world's population, with women having three to five times higher prevalence (1), and costs estimated at €173 billion in Europe (2). In this population, 0.4%–4.4% suffer from cervicogenic headaches (CGH), 1%–4% from tension-type headaches (TTH), and 12% from migraine, with an increase in individuals between 30 and 44 years old (3, 4). Other less common types of headaches exist, including a new daily persistent headache (*N*DPH), hemicrania continua, medication overuse headache and chronic cluster headache (1). While cluster headaches are typically episodic, a chronic form also exists and should be considered in the broader classification of chronic headache disorders (1).

Chronic headaches can significantly impair patients' ability to work and manage stress, leading to reduced productivity, increased absenteeism, and lower quality of life (5). CH are linked to an average of 10 lost workdays over three months and significantly higher rates of long-term sick leave and unemployment, along with markedly reduced quality of life, highlighting the substantial socioeconomic burden of chronic headache (5).

Chronic headaches refers to persistent headaches rather than a specific medical entity (1). The International Headache Society classified patients with more than 15 monthly episodes for three months as chronic daily headaches (CDH). Common characteristics of TTH are bilateral and non-pulsatile. They also lack associated symptoms with tenderness to the pericranial area (1). In contrast, standard features of chronic migraine and CGH include unilaterality, pulsatile severity, moderate to severe pain, and the possible presence of an aura (1). The difference between both is that migraine presents nausea, vomiting, photophobia, and phonophobia more frequently than CGH. Thus, unilaterality is required in migraine as unlocked while locked in CGH (1, 6).

Genetics can contribute to the development of CH. Studies have demonstrated the influence of genetic factors in establishing a distinct “headache threshold.” It can be due to a single gene or multiple genetic variants (7, 8). The environment can also affect CH, such as barometric pressure, air quality, odours, lights, or bright sunlight (9). Additional factors, such as disrupted sleep, obesity, and excessive caffeine intake, can further increase the likelihood of developing CH (1). Medication is an essential part of CH management and may include analgesics, NSAIDs, triptans, antidepressants, anticonvulsants, and muscle relaxants, depending on the headache type and patient profile. However, excessive or prolonged use of these drugs, particularly painkillers, can lead to medication overuse headache where the treatment exacerbates the headache itself (10). Another concern is the growing overuse of botulinum toxin (botox), often promoted as a quick solution on platforms like YouTube or social media (11). Although botox may benefit some chronic migraine cases, its widespread use without adequate clinical assessment risks overtreatment and may reflect public misinformation rather than evidence-based care (11, 12).

Several outcome measures exist to diagnose CH. Initially, it is essential to establish a comprehensive patient profile, encompassing demographic details and more precise information related to the nature of the headache (pain onset, intensity, localization, frequency, evolution, aggravating/easing factors, medication). The subsequent CH diagnosis phase involves administering laboratory tests and brain imaging (1). Blood counts are valuable in detecting infections, and magnetic resonance imaging, the preferred imaging method, gives information concerning possible structural defects. Other tests can help identify CH, like positron emission tomography (PET) scan, magnetic resonance spectroscopy (MRS), and biopsy (1). In case of potential central nervous system infection or idiopathic intracranial hypertension, lumbar puncture may be necessary for diagnostic purposes. Thus, the International Classification of Headache Disorders (ICHD II and III) can also help to classify headaches (1).

Multiple treatments exist for migraine, CGH, and TTH, including pharmacological treatment, cognitive therapies, and physiotherapy, with treatment effectiveness differing according to the headache type (1). Nonpharmacological treatments have shown to be more beneficial for patients presenting TTH, as it is more related to musculoskeletal neck impairments, anxiety, or medication overuse. Traditional physiotherapy interventions include manual therapy, massage, and stretching exercises, which aim to reduce muscle tension and improve mobility. Massage therapy, in particular, has shown positive outcomes in myofascial TMD pain relief (13). Alternative approaches such as biofeedback have been explored for regulating masticatory muscle activity, promoting relaxation, and reducing chronic pain (14).

Several physiotherapy treatments are possible for headaches, and are intended to alleviate pain, improve mobility, or enhance muscle strength to reduce headache frequency and improve quality of life (15). Spinal manipulations or mobilisations have demonstrated effectiveness for CGH, with limited evidence in migraine and TTH. Soft tissue therapies, including compression, strokes, and myofascial trigger point interventions, offer a viable approach for TTH. Finally, cervical spine exercises have exhibited positive outcomes in the cases of TTH, CGH, and migraine. Dry needling is also a potential option for TTH and migraine (16).

Additionally, morphological changes in masticatory and cervical muscles may contribute to headache reproduction (17). Individuals with CH may exhibit reduced muscle thickness, increased asymmetry, and altered activity in muscles such as the masseter, temporalis, sternocleidomastoid, and upper trapezius. These musculoskeletal alterations are particularly relevant in patients with coexisting TMD, where cervical dysfunctions often exacerbate headache symptoms (17). These changes may reflect not only local dysfunction but also central sensitisation, where the nervous system amplifies pain signals, and somatization, while psychological distress contributes to physical symptoms. Such mechanisms may help explain the persistence and severity of pain beyond structural findings (18).

Currently, studies about the relationship between headaches and temporomandibular disorders (TMD) have emerged, demonstrating a direct connection between TMD and headaches (19–21). Recent studies have highlighted several important factors influencing TMD and headache comorbidity. Sleep bruxism, cancer history, and gastroesophageal reflux disease significantly impact pain and headache severity in TMD patients (22). Psycho-emotional factors, such as worsened sleep quality, insomnia, and daytime sleepiness, are strongly associated with orofacial pain and headache perception (22).

Temporomandibular disorders are a chronic condition characterized by persistent, spontaneous pain unrelated to dental issues, occurring in the masticatory muscles, periauricular region, teeth, and temporomandibular joint (TMJ) (19). Typical symptoms encompass TMJ pain, mandibular movement limitation, and TMJ noises (23). Orofacial pain, defined as pain in the face and oral cavity, is also a common symptom of TMD (24). Research has revealed an increased prevalence of CH in individuals with TMD (19, 25).

X-rays, magnetic resonance imaging, and computed tomography scans are additional assessment tools that can also be used. All these outcome measures can help to assess joint effusion, disc displacement, soft tissues, the state of the dentition and joints, or even look at severe joint degeneration, fractures, and dislocations (23). Finally, the Research Diagnostic Criteria for TMD (RDC/TMD) is a valuable outcome that provides multiple information to distinguish TMD and diagnose specific TMD subtypes (26).

Treatment of temporomandibular disorders through physiotherapy encompasses a range of interventions. These include assessing and managing muscle spasms in the TMJ muscles such as masseter, temporalis, internal pterygoid, external pterygoid, sternocleidomastoid, upper trapezius, splenius, and semispinalis. Additionally, the therapeutic approach involves techniques like head and neck massage, addressing trigger points, and implementing passive-active and active stretching exercises (27). Healthcare practitioners may also employ treatments like mandibular or cervical mobilisations. Additionally, they may implement exercises to reduce joint noises, correct deglutition, enhance TMJ symmetry and coordination, and improve mouth opening. Postural exercises, specifically targeting the upper body, aiming to improve muscle control at the craniofacial level should also be considered (27). Finally, the role of education is substantial in reshaping patient habits, improving adherence, and instigating lasting behavioural change (28).

This review focuses on the effectiveness of physiotherapy interventions for TMJ disorders in patients with CH, a population often affected by both conditions. While studies have examined TMD and headache separately, few have addressed their intersection, particularly in the context of non-pharmacological treatments like physiotherapy. By synthesizing existing literature, this review aims to provide insights into the benefits of physiotherapy for pain management and improving the quality of life in individuals with both TMD and CH. Given the high prevalence and impact of these conditions, this review is timely and valuable in contributing towards evidence-based clinical practice.

The primary objective of this systematic review is to evaluate the effectiveness of physiotherapy interventions for treating TMJ disorders in individuals suffering from chronic headaches CH and TMD. This review will specifically examine the impact of physiotherapy treatments on pain management, mobility, and quality of life for patients with these conditions. The secondary objectives are to assess the effects of specific physiotherapy interventions, such as manual therapy, cervical exercises, and static stretching, on headache frequency and intensity in patients with TMD. Additionally, the review will explore the variability in treatment protocols and outcome measures across the studies to understand the range of approaches used and their potential implications for treatment effectiveness.

## 2 Materials and methods

This literature search identified studies using TMJ physiotherapy as a treatment for CH to evaluate their efficacy in alignment with evidence-based practice. An electronic search was conducted using three databases for medical research. Searches were conducted in January 2025 in the PubMed, PEDro, and Cochrane databases, using the PICOS strategy (population, intervention, comparison, outcomes, and study design). The following searches were used: Temporomandibular physiotherapy in headaches; Temporomandibular physiotherapy in chronic headaches; Headaches and myofascial pain management; temporomandibular disorders treatments for migraine. The following MeSH terms were used and combined as follows: (Temporomandibular physiotherapy OR Myofascial pain management OR Physiotherapy Reeducation OR Fascial Pain AND Temporomandibular Joint Disorders OR Headache OR Headache Disorders OR Migraine Disorders). To focus on recent and clinically relevant evidence, the search was limited to studies published from 2010 through January 2025.

Although major medical databases were searched (PubMed, PEDro, and Cochrane), we acknowledge that the exclusion of other databases such as Embase, Scopus, or Web of Science may have limited the comprehensiveness of the review and led to the omission of potentially relevant studies. In addition, while the review protocol was not registered in PROSPERO, it was developed and agreed upon by all authors before the literature search to ensure methodological consistency and rigour. Nonetheless, we acknowledge that the lack of registration may limit the transparency of the review process and potentially introduce reporting bias, in addition to potentially resulting in replication of this study by other researchers. We reported the systematic review according to the guidelines presented in the PRISMA 2020 Statement (29).

### 2.1 Study selection

**Figure 1:**
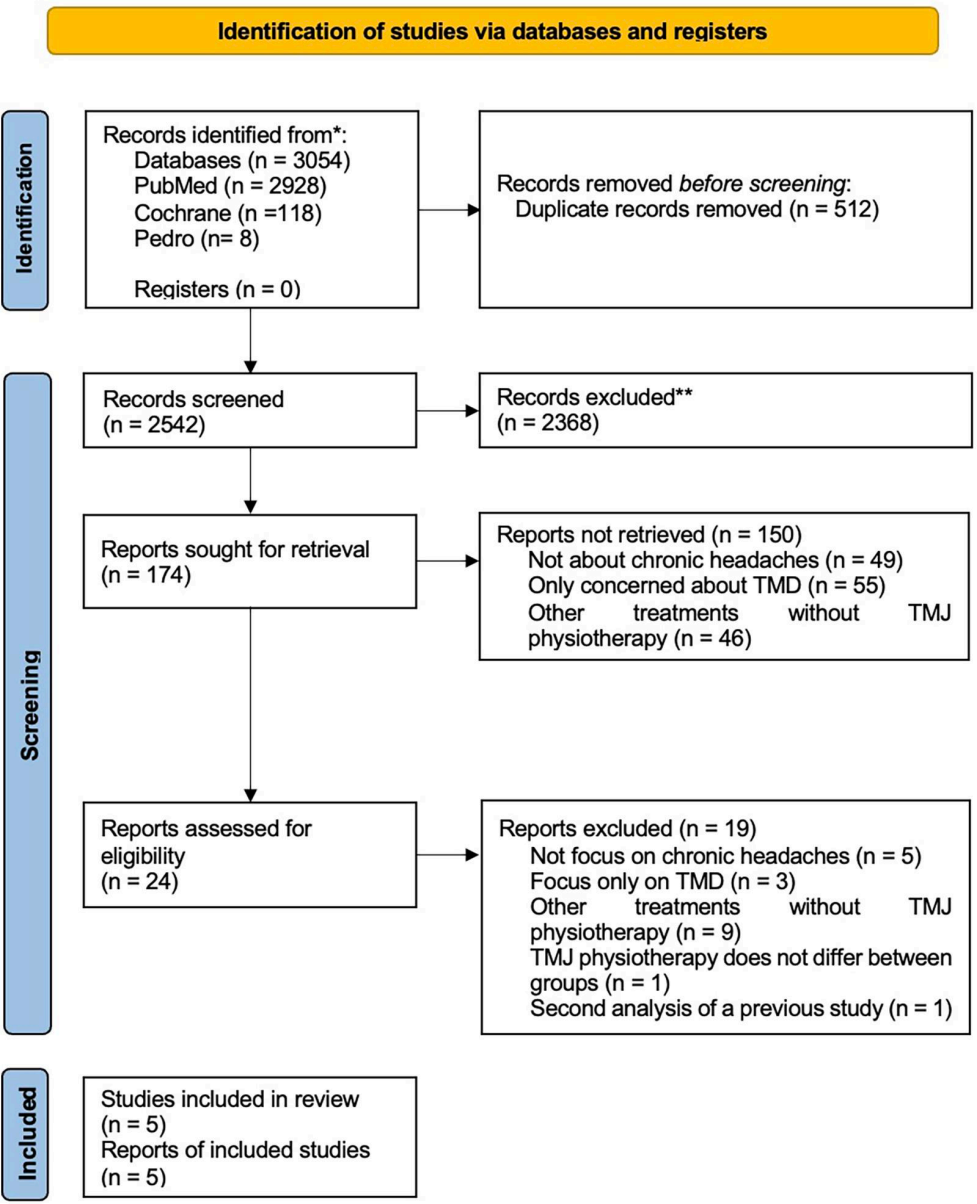
Preferred reporting items for systematic reviews and meta-analyses (PRISMA) flow chart.

The search yielded 2,865 papers from 1954 to 2023 (Table 1). Two reviewers (CQ and JL) independently screened the titles and abstracts to identify relevant studies. The two reviewers independently reviewed full texts, and a third reviewer (KB) was used to resolve the disagreement related to inclusion. The study ultimately included five randomized controlled trials (RCT), with the search summarized in Figure 1.

**Table 1:** Inclusion and exclusion criteria.

Eligibility criteria		Justification
Inclusion criteria	Patient with chronic TMD and headaches (> 3 months)	Population of interest for the research.
	Research involving physiotherapy for the TMJ: - Patient education - TMJ mobilizations/manipulation - Posture training - Cervical spine mobilization - TMJ muscle spasms and trigger points treatments - Stretching (cervical spine, mandibular muscles) - Proprioceptive exercises for TMJ - Technique to improve deglutition (27)	The literature subject is about temporomandibular physiotherapy
	Research involving at least one of the following outcome measures: - Headache Impact Test 6 - Pain scale (Visual Analog Scale, Numeric Pain Rating Scale, and Colored Analog Scale) concerning headache intensity - Craniofacial Pain and Disability Inventory (30–34)	
	Articles available in English, Spanish, and French	Extend the scope of available articles.
	Full-text available	To have complete access to the text
	Publication from 2010 to 2023	To focus on recent studies
	Randomized controlled trials	Enhance studies evidence
Exclusion criteria	Patients with pathologies other than chronic headaches	Not the population that will be studied in the literature
	PEDro score below 5/10	To guarantee the study quality
	Studies involving treatments without TMJ physiotherapy	Narrow the research on physiotherapy treatments

### 2.2 Data collection

A single reviewer (CQ) carried out the data extraction process. Subsequently, a second reviewer (JL) examined the extracted data to ensure accuracy. An analysis of each study's characteristics was performed. Data encompassed the author's name, publication year, study type, number of participants, and specific participant characteristics (sex, mean age, CH/TMD diagnosis). Comprehensive records regarding the interventions employed in each study were also available. These records consisted of a wide range of data, including the types of interventions employed, the frequency of these interventions (session/week), and the total duration of the interventions. Details on the outcome measures used in the studies and their corresponding results were also recorded.

### 2.3 Risk of bias assessment

The Revised Cochrane tool for evaluating the risk of bias in randomized trials (RoB2) was employed for critical appraisal to assess the studies' risk of bias. This tool comprises five bias domains, encompassing 22 signaling and optional questions. Responses to the questions include “yes,” “probably yes,” “probably no,” “no,” and “no information,” which result in categorizations of “low,” “some concerns,” or “high” risk of bias. The tool is based on individual questions, leading to subjective interpretation as there is no numerical scoring system (35). The risk of bias was assessed based on the randomization process, the deviations from intended interventions, the missing outcome, the measurement of the outcome, and the selection of the reported results (35).

### 2.4 Characteristics of the studies

The data extraction for each study is shown in Table 2. The studies includes five RCTs and a total of 462 participants aged between 18 and 63 years old (36–40). Of the five included studies, three incorporated both men and women, while two included only women (36, 38). All studies were available in English. Two studies explored the effects of orofacial treatment on people with chronic TMD and headaches compared to standard care (37, 40). One study examined the benefits of the upper cervical region and craniocervical flexor training vs. no intervention (36). Another study looked at the impact of global postural re-education (GPR) vs. static stretching (SS) for individuals with TMD (38), while a final study compared the effectiveness of educational interventions vs. occlusal splints for patients with chronic TMD (39).

**Table 2:** Data extraction tables of the included studies.

Author, (Date) country, city settings	Design	Aim of the study and participant information	Intervention information	Outcome measures	Reported results
Calixtre et al., 2019 (36)The Federal University of de São Carlos—UFSCar	Single-blind RCT	Aim: Determine whether mobilization of the upper cervical region and craniocervical flexor training decreased orofacial pain, increased mandibular function and PPT of the masticatory muscles, and decreased headache impact in women with TMD compared to no intervention.Description: *N* = 104 (18–40 years) - Participants excluded (*N* = 43) due to non-meeting the inclusion/exclusion criteria ***N* = 61***Randomization was done using opaque envelopes (sealed and numbered) prepared by one of the researchers not involved in the recruitment or the assessment of the subjects*. 30 patients in the intervention group - *N* = 30 at baseline - *N* = 27 at the end of the study: 1) Lost due to personal reasons (*N* = 1) 2) Lost due to the start of psychoactive drugs (*N* = 2) Mean age: 26.1 Gender: 30F 31 patients in the control group - *N* = 31 at baseline - *N* = 29 at the end of the study: 1) Lost due to personal reasons (*N* = 1) 2) Lost due to the start of psychoactive drugs (*N* = 1) Mean age: 26.3 Gender: 31F - Number of participants included for the final analysis: (*N* = 56) **Inclusion criteria** - Female - Between 18 and 40 years old - Orofacial pain for at least three months (chronic pain according to the IASP - Baseline pain score ≥ 3 on a ten-point NPRS - Diagnosis of orofacial myalgia (Ia and Ib) or mixed TMD of Ia/Ib and groups IIa/IIb/IIIc (disc displacements) and IIIa (TMJ arthralgia according to RDC for TMD **Exclusion criteria** - Pregnancy - Diagnosis of fibromyalgia or rheumatic or neurologic issues - History of neck or jaw fracture - Dental loss (except third molars, when extracted > six months ago) - Previous orofacial treatment (orthodontics or physiotherapy in the previous six months) - Occlusal splints or regular medication for less than six months (not exclude if > six months, but exclude if starting new treatment)	Description Frequency: twice a week—20 min Duration: five weeks Total: 10 sessions **Intervention group:** - Face to face treatment—individual - Nonmanipulative manual techniques, neck motor control/stabilization exercises with biofeedback: 1) Suboccipital inhibition technique (for 2 min) 2) Passive anterior-posterior upper cervical mobilization (3 × 2 min, with 30 of rest = seven minutes) 3) Sustained natural apophyseal glide mobilization with rotation on C1-C2 vertebras (10 times to each side) 4) Craniocervical flexor stabilization exercise (maintain pressure for 10 s with no contraction of superficial neck flexor muscles—10 times) *A physiotherapist with five years of experience in the musculoskeletal disorders (PT1) delivered the treatment:* **Control group:** - No intervention or counseling for five weeks	*Another physiotherapist (PT2), blinded to the allocation, performed the following outcome measures at baseline and five weeks:* - Baseline demographic and diagnostic characteristics of each group (Age, BMI, Years of pain, Orofacial pain with NPRS, NDI, MMO, Headache, Splint therapy) **Primary outcome:** 1) Orofacial pain (using VAS): - Current orofacial pain at the evaluation - Maximum orofacial pain in the last week - Minimum orofacial pain in the last week **Secondary outcome:** - PPT of masticatory muscles (using digital algometer) - HIT-6 - MFIQ	Baseline characteristics did not differ significantly between the two groups (*P* ≥ .05)**Primary outcome:** **Orofacial Pain:** 1) Current pain: - Significant group-by-time interaction (*P* < 0.01) - Significant within-group effect for the IG group, while not for the CG - Significant difference between groups at five weeks follow-up: within-group ES for the IG and the between-groups ES were large -- ES of CG was null 2) Maximum pain - Significant group-by-time interaction (*P* < 0.01) - IG showed significant within-group improvement, while not for CG - Significant between-group difference at five weeks follow-up: within-group ES was significant for the IG and trim for the CG. - The between-groups ES was moderate, as IG experienced less pain than CG. 3) Minimum pain - Significant group-by-time interaction (*P* = 0.03) - Significant within-group difference for the IG, while not for CG - Significant between-groups difference at five weeks follow-up. ES within-group for the IG and the between-groups ES were both moderate (< 0.50) - ES for CG was null Weekly average scores of the three pain intensity measures: - Significant interaction between time and group (*P* < 0.01) - Within-group: mean pain intensity scores from IG in week 4 (*P* < 0.01) and week 5 (*P* < 0.01) differed from baseline (*P* < 0.01) - No differences from baseline for CG - Significant between-group difference from the fourth week of the protocol **Secondary outcome** 1) **PPT:** - Little variation in the between groups and over time - No significant group-by-time interaction (*P* = 0.58) - No main effects of time and between groups - ES irrelevant 2) **HIT-6:** - Significant group-by-time interaction - Significant within-group difference for the IG, while not for CG (*P* = 0.09) - Significant between-groups difference at follow-up - Within-group ES for the IG and the between-groups ES were significant (> 0.85), favoring intervention. - ES for CG small 3) **Mandibular function:** - Significant group-by-time interaction for mandibular function (*P* = 0.02) - Significant between-groups difference at follow-up - IG: significant within-group difference - CG: no significant difference from baseline to follow-up (*P* = 0.93) - Within-group ES for CG small - ES for the IG and the between-groups ES: Moderate
Garrigós-Pedrón et al., 2018 (37)Department of NeurologyHospital Universitario Miguel ServetZaragoza, Spain	RCT	Aim: Analyze the effects of adding orofacial treatment to cervical physical therapy in patients with chronic migraine and TMD.Description*N* = 65 (18–65 years) - - Participants excluded (*N* = 13): 1) Due to non-meeting the inclusion criteria (*N* = 5) 2) Decline to participate (*N* = 8) ***N* = 52** *Randomization was done using a randomized computer program (randomization.com), grouped according to age and sex, and assigned by a study member blinded*. 26 patients in the cervical group - *N* = 24 patients at baseline (*N* = 2 withdrawn due to incompatible schedule) - *N* = 22 patients in follow-up: 1) Lost due to pregnancy (*N* = 1) 2) Lost due to surgical intervention (*N* = 1) Mean age: 48.2 Gender: 19F—3M 26 patients in the cervical and orofacial group - *N* = 25 patients at baseline (*N* = 1 withdrawn due to incompatible schedule) - *N* = 23 patients in follow-up: 1) Lost due to death in family (*N* = 1) 2) Lost due to nonadherence to treatment (*N* = 1) Mean age: 46.0 Gender: 20F—3M Number of participants included for the final analysis (*N* = 45) Gender: 39F- 6M **Inclusion criteria** - Patients diagnosed with chronic headaches by a neurologist specialized in headaches based on the criteria of the ICDH-III - Myofascial TMD according to the RDC/TMD **Exclusion criteria** - TMD due to disc displacement - Osteoarthritis - Inflammatory arthritis of TMJ - Other chronic diseases (Respiratory, cardiovascular, and MSK disorders such as chronic polyarthritis, rheumatic muscular inflammation, osteoporosis, and osteoarthrosis) - Other headaches, neurologic diseases, or dental problems - Cognitive, emotional, or psychological disturbances - Previous surgery or trauma in the orofacial region - Orthodontic or physical therapy treatment in the last six months	Description Frequency: 30 min/session Duration: three-to six-week period Total: six sessions **Cervical group** - Treatment only in the cervical region - Explanation on how to perform exercises, sets, repetitions, rest periods, frequency, and general mistakes) - Manual therapy - Several techniques (Suboccipital muscle inhibition, cervical joint, passive mobilization in supine and prone positions, co-contraction of flexors and extensors, nerve tissue techniques (3 × 10 repetitions) - Self-care tips (position of the head during the day, avoid working with head tilted, maintain good cervical ergonomics) - Home exercises explained and practiced in consultation once a day for five days **COG group:** - Cervical and additional orofacial region treatment - Explanation on how to perform exercises, sets, repetitions, rest periods, frequency, and general mistakes) - Several techniques: a longitudinal caudal bilateral technique in the TMJ, neuromuscular technique in the masseter, frontal muscles, and coordination exercises of the masticatory muscles (3 × 10 repetitions for each exercise) - Self-care tips (avoid eating hard food, avoid maximum mouth opening, no chewing gum, no sleeping on the affected side, yawning with the tongue in the upper incisors, and keep the tongue in the upper incisors. - Home exercises explained and practiced in consultation, once a day for five days Both groups: - Continued their medication - Could not withdraw from pharmacologic treatment during the study - All had a similar intake of routine medication with continuous preventive treatment and abortive pharmacological treatment at the onset of migraine attacks *The treatment techniques were applied by the same physiotherapist (P.N.D) who had > three years of clinical experience in craniofacial techniques.*	**Primary outcomes:** - Sociodemographic questionnaire: age, sex, height, weight, duration of pain, educational level, and work status - CF-DPI - HIT-6 **Secondary outcomes:** - TSK-11 - Pain intensity measured on a VAS - PPT in the temporal, masseter, and extra trigeminal (wrist) region, bilaterally (Wagner instruments) - The pain-free MMO A blind investigator performed all outcome measures at: - Pre-treatment - Post-treatment - Six weeks after the final treatment (follow-up 1) - 12 weeks after the final treatment (follow-up 2) - investigator *The assessor was blinded to the subject's group assignments*.	**Significant differences for the group × time interaction:** - CF-DPI (*P* = 0.027) - HIT 6 (*P* < 0.001) - Pain Intensity (VAS) (*P* < 0.001) - PPT in the temporalis muscle (T1) (*P* < 0.001) - PPT on the origin of the masseter muscle (M1) (*P* < 0.001) - PPT on the insertion of the masseter (M2) (*P* < 0.001) - Pain-free MMO (*P* < 0.001) **No significant differences for the group × time interaction:** - TSK-11 (*P* = 0.023) - Extra trigeminal region (*P* = 0.55) **Significant differences between baseline and posttreatment:** - CF-DPI: COG group (*P* = 0.001) - HIT-6: cervical group (*P* < 0.001) - PPTs: COG group (T1: *P* < 0.001; M1: *P* = 0.001, M2: *P* < 0.05) **No significant differences between baseline and posttreatment:** - CF-DPI: cervical group (*P* > 0.05) **Significant differences between baseline and follow-ups 1 and 2:** - CF-DPI: cervical group (*P* < 0.05) - CF-DPI: COG group (*P* < 0.001) - HIT-6: cervical group (*P* < 0.05) - M2: COG group (*P* < 0.001) **Significant differences between baseline and follow-up 1:** - T1: COG group (*P* = 0.002) - M1: COG group (*P* = 0.018) **Significant differences between baseline and follow-up 2:** - T1: COG group (*P* < 0.001) - M2: COG group (*P* < 0.001) **No significant difference between baseline and follow-up 2:** - VAS: cervical group - Significant differences between baseline, posttreatment, and follow-ups 1: - VAS: cervical group (*P* < 0.05) **Significant differences between baseline, posttreatment, and follow-ups 1 and 2** - HIT-6: COG group (*P* < 0.001) - VAS: COG group (*P* < 0.001) - Pain-free MMO: COG group 2 (*P* < 0.001) **Significant differences between groups in posttreatment and at follow-ups 1 and 2** - Pain Free MMO: posttreatment (*P* = 0.14); follow up 1 and 2 (*P* < 0.001) **Significant differences between groups at follow-up 2:** - CF-DPI (*P* = 0.042) - HIT 6 (*P* = 0.002) - VAS - Three trigeminal points (*P* < 0.05) **No significant differences over time:** - T1: cervical group (*P* > 0.05) - M1: cervical group (*P* > 0.05) - M2: cervical group (*P* > 0.05) - Pain-free MMO: cervical group (*P* < 0.05)
Maluf et al., 2010 (38)Clinical Evaluation and Intervention Laboratory of the Department of Speech, Physical Therapy, and Occupational Therapy of the University of São Paulo.	RCT	Aim: Compare two different exercise interventions, GPR and SS, for treating TMD symptoms and assess PPT and EMG activity of several muscles in women with myogenic TMD. Description: ***N* = 28** (19–40 years old) *Randomization was done using opaque envelopes*. 14 patients in the global posture reeducation group (GPR) - *N* = 14 at baseline - *N* = 12 at the end of study: 1) Lost due to work-related reasons (*n* = 2) - *N* = 12 at follow-up Gender: 14F Mean age: 30.0 14 patients in the static stretching group (SS) - *N* = 14 at baseline - *N* = 12 at the end of study: 1) Lost due to work-related reasons (*n* = 2) - *N* = 12 at follow up Gender: 14F Mean age: 30.08 - Number of participants included for the final analysis: (*N* = 24) **Inclusion criteria** - Chronic pain (> 3 months) - Helkimo index III - Myogenic TMD - Presence of parafunctional habits (bruxism, teeth clenching, mouth breathing, and lip biting) - Masticatory myofascial pain according to the revised criteria of the RDC/TMD **Exclusion criteria** - Surgery or trauma in the orofacial region - Systemic or degenerative diseases in the spine and upper limbs - Undergoing odontology, psychologic, or physical therapy treatments	Description Frequency: 1 individual session/week—45 min Duration: 8 weeks Total: 8 sessions First 10 min of sessions: - Patients rested (in supine position with all limbs relaxed) - Manual therapy maneuvers made as described by Bienfait - Breathing exercises to stretch the fasciae that recover the shoulders, as well as the cervical spine muscles - Stretching treatment for 30 min **GPR group:** - Patient maintained free breathing, with no breath-holding - At each session, patients maintained two different postures (15 min each) - To stretch the posterior muscle chain: Patients positioned in the supine position to achieve the final stretching position with adducted upper limbs and lower limbs at 90° hip flexion supported by a hanging strap - Gradual knee extension progressively performed until tolerated, with the ankle in dorsal flexion, keeping the occipital, lumbar region, and sacrum stabilized, as rectified as possible - Anterior muscle chains stretched with the patient in the supine position and upper limbs abducted at 30° with supine forearms - Pelvis kept in retroversion whereas lumbar spine remained stabilized - Hips flexed, abducted, and laterally rotated, with the soles of the feet touching each other - Lower limbs extend, maintaining a 90° tibiotarsal angle, toes relaxed, and lumbar region on the table. At the end, the arms reach 140° abduction. **SS group:** - Patients performed static stretching exercises for the cervical spine, head, upper limbs, and mandibular muscles (masseter and anterior temporalis) - Each stretching position held for 30 s, keeping a slow breathing rhythm and avoiding compensations. - Exercises bilaterally repeated three times after a 10-second rest pause - Patient's limits and possibilities considered All interventions were performed by an experienced investigator previously trained and blinded.	**Primary outcome:** 1) Demographic and clinical characteristics (Age, mandibular depression, occupation, and cervical alignment) 2) Symptoms and Pain Intensity (VAS-10 cm horizontal line) with symptoms: - Pain at TMJ - Headache - Cervicalgia - Teeth clenching - Ear symptom - Restricted sleep - Difficulties with mastication **Secondary outcome:** - PPT (algometer) - EMG activity (Fischer, https://www.wagnerinstruments.com, Greenwich, CT) All evaluations were performed by an experienced investigator previously trained and blinded at: - Baseline - After treatment end - At follow-up (8 weeks after treatment)	There are no significant between-group differences for age and mandibular depressionCervical rectification was observed in 50% of patients **Primary outcome:** 1) **Symptoms and VAS-10 cm horizontal line:** - At the second evaluation: statistically significant decrease (*P* < 0.05), except for restricted sleep and restricted mastication (both groups), and ear symptoms (GPR) - In the second evaluation, when comparing both interventions, there was a significant decrease in headache (*P* < 0.024) - At the third evaluation, pain at TMJ, headache, and teeth clenching were significantly improved (vs. baseline) for both groups - At the third evaluation, cervicalgia was reduced in the GPR group only (*P* < 0.002) **Secondary outcome:** 1) **PPT** - At the second evaluation, significant improvements for all muscles (*P* < 0.05) - At the third evaluation, values decreased in the SS group, but excepting the masseter muscle (*P* < 0.016), no significant differences - In the third evaluation, there was a significant difference for the GPR group for the anterior temporalis muscle only (*P* < 0.027). - No significant differences were seen when comparing both treatment groups (*P* > 0.05) 2) **EMG activity** - At the second evaluation, significant decreases for masseter, anterior temporalis, and sternocleidomastoid muscles (*P* < 0.05) - At the third evaluation, differences remained significant only for sternocleidomastoid muscle (GPR, *P* < 0.007; SS: *P* < 0.005) - No differences seen between treatment groups (*P* > 0.05)
Michelotti et al., 2012 (39)Clinic for Temporomandibular Disorders and Orofacial Pain of the University of Naples Federico II	RCT	Aim: Compare the effectiveness of an education program with occlusal splint therapy for treating myofascial pain of the jaw muscle over a short period. Description:*N* = 198 (18–53 years) - Participants excluded (*N* = 154) due to non-meeting the inclusion/exclusion criteria: ***N* = 44***Randomization was done using a balanced block randomization:*23 patients in the education group - *N* = 23 at the end of the study Mean age: 31.4 (20–53 years)Gender: 19F–4M 21 patients in the occlusal splints group - *N* = 21 at baseline - *N* = 18 at the end of the study (*N* = 3 lost due to splint's cost) Mean age: 30.3 (18–49 years)Gender: 15F–6M - Number of participants included for the final analysis (*N* = 41) Gender: 32F- 9M**Inclusion criteria** - Patient with myogenous pain and ongoing pain, either recurrent or constant for > three months (diagnostic categories Ia and Ib in the RDC for TMD - Absence of objective evidence of joint pathology or dysfunction - Spontaneous muscle pain > 30 millimeters on a VAS **Exclusion criteria** - Disk displacement with or without reduction (diagnostic II in RDC/TMD) - Arthrogenous TMD with pain or radiographic alterations in the temporomandibular joints (diagnostic category III in RDC/TMD) - Other orofacial pain conditions - Other TMD treatments performed in the preceding three months - Neurological or psychiatric disorders or both - History of current abuse of pain medication - Use of occlusal splint in the preceding year	DescriptionDuration of treatment: three months (at home) **For both groups:** - Indication to follow treatment even if pain-free for three months - The same clinician (S.V.) administered both therapies **Education group:** - General information about self-care of jaw musculature - Home exercise program: habits-reversal technique - Reassurance by explaining the problem, the suspected etiology, and the favorable prognosis for this benign disorder - Explanation of the jaw function and the risk of pain if there is an overuse - Paying attention to jaw muscle activity and avoiding usual oral habits and excessive mandibular movements, in addition to following a soft diet - Keep muscle relaxed by holding the mandible in its postural position (teeth apart) and not in occlusion - Determination of mandibular rest position by asking the patient to pronounce the letter “N” several times and to hold the tongue behind the maxillary incisors, with the lips in slight contact - Request that patients practice what they learned at home and during their daily activities by using visual aids to alert them to tooth contact, as well as holding the mandible in a relaxed position - Information about the relationship between chronic pain and psychological stress - Reinforce compliance and motivation **Occlusal splints group:** - Occlusal splint (Stabilization splint, Michigan): one week to receive the occlusal splint accurately adjusted in the centric occlusion	The assessments were made by a baseline clinician (GI) for both groups (blinded to treatments)**At baseline for both groups:** - Accurate alginate impressions of both arches and an interocclusal record with a wax wafer - History and clinical examination of the patient **At baseline for the occlusal splint group:** - Evaluation of participants to determine any need for adjustment of the device to eliminate local irritation of the soft and hard oral tissues and to adjust the occlusal surface so that mandibular teeth would touch the splint evenly and simultaneously **For both groups at baseline and at three months:** - Pain assessments using 100-mm horizontal VAS: Spontaneous muscle pain, pain during chewing, and headache) - Pain-free MMO	**Baseline characteristics did not differ significantly between the two groups (*P* ≥ 0.05)** **Pain-free MMO:** - No significant difference between treatment groups (*P* = 0.325) at baseline. - Significant difference over time (*p* = 0.001) - No significant difference between the two groups (interaction × treatment group; *P* = 0.528) **VAS score for spontaneous muscle pain:** - No significant difference between groups (*P* = 0.623) - No significant difference across time (*P* = 0.197) **Spontaneous muscle pain score:** - Significant difference between groups (interaction × treatment group; *P* = 0.034) - Significant difference across time in the education (*P* = 0.017) but not in the occlusal splint group (*P* = 0.540) **Pain during chewing and Headache scores:** - No significant difference for time, treatment, group, and effect of treatment (*P* ≥ 0.106)
von Piekartz and Ludtke, 2011 (40)Netherlands	Single-blind RCT	Aim: 1) Identify the prevalence of TMD in a sample of patients diagnosed with cervicogenic headaches 2) Determine the tests that are clinically relevant to detect TMD in CGH patients 3) Evaluate the effect of additional orofacial physical therapy after three and six months in comparison with a control group Description:*N* = 67 (18–65 years) - Participants excluded (*N* = 24) 1) Due to non-meeting TMD inclusion criteria (*N* = 5) 2) Due to non-meeting other inclusion criteria (*N* = 1) 3) Decline to participate (*N* = 18) ***N* = 43** Gender: 27F–16M Mean age: 36 *Randomization was done by a third researcher using a computerized random number generator*. 21 patients in the usual care group - *N* = 21 at baseline - *N* = 18 at the end of study: 1) Lost due to an increase in symptoms (*N* = 2) 2) Lost due to falling downstairs (*N* = 1) - *N* = 18 at follow-up 22 patients in the orofacial care group - *N* = 22 at baseline - *N* = 20 at the end of study: 1) Lost due to an increase in symptoms (*N* = 1) 2) Lost due to death in family (*N* = 1) - *N* = 20 at follow up - Number of participants included for the final analysis (*N* = 38) Gender: 25F-13M (18–63 years) Mean age: 32 **Inclusion criteria** - CGH diagnostic according to ICDH-II - Headache > 3 months - No prior treatment for TMD - NDI score > 15% - At least one of the four signs of TMD based on previously reported criteria: 1) Joint sounds 2) Deviation during mouth opening 3) Extraoral muscle pain at a minimum of two tender points in the masseter or temporalis muscle 4) Pain during passive mouth opening **Exclusion criteria** - Orthodontic treatment in the past	**Description** Frequency: 1 individual session/week −30 min Duration: 3–6 weeks (21–42 days) Total: 6 sessions **Usual care group:** - Continued their treatment of the craniocervical region, the therapist selected the technique and treatment or exercise type that he considered beneficial **Orofacial group:** - Accessory movement of the temporomandibular region - Masticatory muscle techniques, such as tender-trigger point treatment and muscle stretching - Active and passive movement, facilitating optimal function of cranial nerve tissue - Coordination exercises - Home exercises - Address Masticatory trigger points, muscle tightness, and temporomandibular joint restriction - Technique to desensitize cranial nerve tissue when necessary - Home exercises, individualized to the patient - Additional neuromusculoskeletal treatment of the cervical region, if necessary *Three specialist manual therapists with at least 4-years of experience managing orofacial pain take care of the orofacial treatment group*.*The four treating therapists in the usual care group were primary contact practitioners with > five years of work experience. They completed a manual therapy training program recognized by the International Federation of Orthopedic Manual Therapy (IFOMPT).*	**Primary outcome:** - CAS—VAS **Secondary outcomes:** - NDI - AQ - Noise Registration at the Mandibular Joint - GCPS - Mandibular Deviation - Mouth Opening Measurement - PPT Measurement of the masticatory muscles using Algometry - VAS: mouth opening and for headache intensity A blinded investigator with IFOMT-level training and five years of post-graduate experience performed the three assessments of all measures. - Before the first treatment - After six treatments (within 4–6 weeks) - After six months of follow-up *Prior to treatment: The UCG group received an orofacial examination from the treating physical therapist.*	No significant difference in age, gender, and duration of the complaints between UCG and OFG. **Primary outcome:** 1) **CAS:** - At baseline: no significant difference between UCG and OFG (*P* < 0.05) **Secondary outcomes:** 1) **AQ:** - Between the second and third measurement: significant decrease in OFG 2) **NDI:** - Third measurement: no significant difference in OFG **For CAS AND AQ** - After six treatments, average of AQ of OFG decreased by more > 50% - Second and third assessment: significant difference between the groups (*P* < 0.001), with a significant reduction observed in OFG compared to UCG that increases and leads to a possible deterioration of headache complaints **For CAS, NDI, AQ** - Second measurements: significant difference between the groups, with decreased OFG compared to UCG (*p* < 0.001) - Second and third measurements: no significant difference in UCG compared with the first measurement (*P* > 0.05), and significant difference in OFG compared with the first measurement (*P* < 0.001) - Second and third assessment: significant difference between the groups (*P* < 0.001), with a reduction observed in OFG compared to a significant increase in UCG 3) **GCPS** - At first measurement: average score in UCG and OFG - After the third measurement, scores in percentages in the grade II and III groups were reduced in OFG and slightly increased in UCG - From first to third measurement: OFG shifted in grade I, with no improvement in UCG 4) **PPT** - No significant difference (*P* < 0.05) for 10 out of 12 examined tender point regions. Only significant difference for left and right anterior temporal muscle (*P* > 0.001) - Second measurement: significant difference between the UCG and OFG groups, with an improvement in OFG. - Second and third measurement (PTM from anterior masseter muscle): no significant differences in UCG (*p* > 0.05) compared to the first measurement 5) **TMD signs** (mouth opening, pain and range, deviation, sounds, and PPT of the anterior temporal muscles) - Second and third measurements: no significant difference in UCG compared with the first measurement - Second measurement: significant difference between groups - Second and third measurements: Trend towards decrease in OFG compared to UCG 6) **VAS** - 85% of patients in OFG present the following criteria, while 0% in UCG: - Mouth opening range (>5 mm), decrease in pain intensity (>22 mm), NDI (>3,5), and VAS for headache (>20 mm) 7) **Mouth opening:** - No significant difference in both groups but slightly improvement in OFG - Second and third measurements: no significant difference in UCG compared with the first measurement (*P* > 0.05), and significant difference (*p* > 0.001) in OFG - Third measurement: no significant difference in OFG

AQ, Anamnestic questionnaire BMI, Body Mass Index; CAS, Colored analog scale; CF-DPI, Craniofacial pain and disability inventory; CG, control group; CGH, Cervicogenic headaches; COG, Cervical and orofacial group; EMG, Electromyographic; ES, Effect sizes; GCPS, Graded chronic pain status; GI, Baseline clinician; GPR, Global postural reeducation; HIT-6, Headache impact test 6; IASP, International association for the study of pain; ICDH, International classification headache disorders-III; IFOMPT, International federation of orthopedic manual therapy; IG, Intervention group; M1, Origin of the masseter muscle; M2, Insertion of the masseter muscle; MFIQ, Mandibular function impairment questionnaire; MMO, Maximal mouth opening; MSK, Musculoskeletal; NDI, Neck disability index; NPRS, Numerical pain rating scale; OFG, Orofacial group; P.N.D, Physiotherapist; PPT, Pressure pain threshold; PT1, Physiotherapist with five years of experience in the musculoskeletal disorders; PT2, Physiotherapist blinded to the allocation; RCT, Randomized controlled trials; RDC for TMD, Research diagnostic criteria for temporomandibular disorders; SS, Static stretching; SV, First clinician; TMD, Temporomandibular disorders; TMJ, Temporomandibular joint; TSK-11, Tampa scale for kinesiophobia 11; T1, Temporalis muscle; UCG, Usual care group*.*

The follow-up period varied, ranging from five weeks to six months. Three articles used the Visual Analogue Scale (VAS) to measure the intensity of headaches (38–40), while two others utilized VAS to assess pain intensity (37) and orofacial pain (36). The VAS was also used to measure pain during mouth opening (40). Two articles used the Headache Impact Test-6 (HIT-6) to evaluate the adverse effects of headaches on daily activities (36, 37). Additionally, one article used the Colored Analog Scale (CAS) to rate the intensity of headache pain (40). Finally, one researcher utilized the Craniofacial Pain and Disability Inventory (CF-DPI) to evaluate headache frequency (37). The intervention frequency differs among the studies, going from one or two sessions per week (36–38, 40) to daily sessions at home over three months (39). The duration of each session varied as well, ranging from a few minutes for home therapy (39) to 20–45 min with a physiotherapist (36–38, 40).

### 2.5 Quality assessment

The risk of bias between studies was variable and is presented in Table 3. Four studies reported an adequate randomization process (36–38, 40) with a low risk of bias, while one study presented some concerns due to “no information” on concealment (39). All the studies present some concerns for the first part of the domain concerning deviations from the intended intervention, meaning that either personnel, participants, or both were not blinded. In addition, no information on the deviations was available (36–40). All studies performed an appropriate analysis, leading to a low risk of bias for the second part of the domain. Concerning the third domain of the RoB2 tool, which is bias due to the missing outcome, all studies present a low risk. All the studies present some concerns about the measurement of outcome, which arise due to no information on the blinding of outcome assessors at the end of the study (36–40). For the last domain, all studies present a low risk of bias concerning the selection of the reported result (36–40).

**Table 3:** Risk of bias of the included studies.

Authors	Randomization process	Deviations from intended intervention	Missing outcome	Measurement of outcome	Selection of the reported outcome
Calixtre et al. (36)	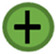	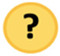 , 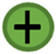	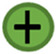	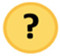	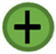
Garrigós-Pedrón et al. (37)	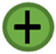	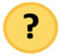 , 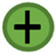	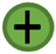	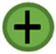	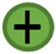
Maluf et al. (38)	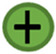	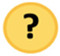 , 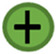	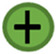	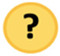	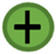
Michelotti et al. (39)	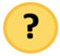	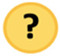 , 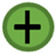	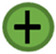	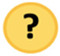	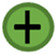
von Piekartz and Ludtke (40)	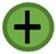	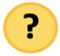 , 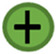	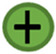	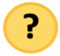	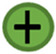

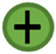
, low risk of bias, 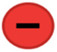, high risk of bias, 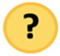, some concerns.

## 3 Result of studies

### 3.1 Orofacial treatment vs. control group with usual care

Two studies (37, 40) compared orofacial treatment with usual care. Even though interventions are not precisely similar, both experimental groups included techniques at the TMJ associated with advice and home exercises. The control group also differs but shows some similarity in the treatment at the craniocervical region, with techniques avoiding the TMJ. Concerning results for headaches (37), Garrigos-Pedron et al. highlighted significant results for the CF-DPI, with differences for the group × time interaction (*p* = 0.027). There were also significant differences between baseline and post-treatment (*P* = 0.001), between baseline and follow-ups 1 and 2 (*P* < 0.001) for the cervical and orofacial group (COG). However, the study also showed significant positive results for the cervical group between baseline and follow-ups 1 and 2 (*P* < 0.05).

Concerning HIT-6, significant differences were present for the group × time interaction (*P* < 0.001). The cervical group had significant differences between baseline and post-treatment (*P* < 0.001) and baseline and follow-ups 1 and 2 (*P* < 0.05). The COG significantly differed between baseline, post-treatment, and follow-ups 1 and 2 (*P* < 0.001). Thus, significant group differences occurred at follow-up 2 (*P* = 0.002) (37).

von Piekartz and Ludtke (40) used the CAS as an assessment of headache pain intensity. Results highlighted significant differences between the groups (*P* < 0.001) at the second and third measurements, with a reduction observed in the orofacial group (OFG) compared to a significant increase in the usual care group (UCG). The average CAS result decreased by more than 50% after six treatments in the OFG (40).

Both studies used VAS to evaluate pain, but only the study of von Piekartz and Ludtke (40) used it for headache pain intensity, with results that showed a decrease superior to 20 mm for 85% in the OFG, compared to 0% in the UCG, with a result of more than 20 mm being significant.

### 3.2 Education vs. an occlusal splint

In the study of Michelotti et al. (39), a comparison occurred between education and occlusal splint interventions. The education group received information, a home exercise program, reassurance, and guidance on TMJ-related practices to enhance compliance and motivation. Conversely, the other group received occlusal splints with TMJ techniques, advice, and home exercises. Headache pain intensity was assessed using VAS, revealing non-significant differences across time, treatment groups, and treatment effects (*P* > 0.106) (39).

### 3.3 Mobilization of the upper cervical region and craniocervical flexor training vs. no intervention

Calixtre et al. (36) compared upper cervical region treatment and craniocervical flexor training to no intervention for the control group (CG). The intervention group (IG) received non-manipulative techniques, neck motor control exercises, and stabilization with biofeedback. The research used HIT-6 for headache evaluation. Results indicated a significant group-by-time interaction, showing a significant within-group difference for the IG but no significant difference for the CG (*p* = 0.09). The intervention group (IG) demonstrated a notable within-group effect size, and between-group effect sizes (>0.85) significantly favored the intervention. Conversely, the CG exhibited a small effect size (36).

### 3.4 GPR vs. SS exercises

This study compared GPR to SS exercises. The GPR group received several treatments concerning posture, while the SS group received stretching (cervical spine, head, upper limbs, and mandibular muscles). Headache pain intensity was measured using VAS. The second evaluation demonstrated a significant decrease in headache pain for the SS group (*P* < 0.024) when comparing both. In the third evaluation, headache pain improved in both groups compared to baseline (*P* < 0.002). However, SS lead to superior improvements (38).

### 3.5 Other results

Concerning other results not directly related to our research, Garrigós-Pedrón et al. (37) showed significant differences for COG at each time and between the cervical group and COG at post-treatment and follow-ups 1 and 2. Regarding pressure pain threshold (PPT), the study revealed significant differences in the COG, with a notable distinction between the cervical group and COG at the final follow-up. Pain intensity also showed improvement for both groups.

In Michelotti et al. (39), significant reductions in spontaneous muscle pain were observed exclusively in the education group over a brief period. Pain-free maximal mouth opening (MMO) did not differ significantly between groups at baseline and between the groups but significantly changed over time. VAS measurements revealed no significant differences, and the same was valid for pain during chewing.

Calixtre et al. (36) demonstrated significant reductions in orofacial pain intensity for the IG. However, there were no significant changes in PPT for the masticatory muscles. Additionally, an improvement in mandibular function was observed, with differences below the minimum detectable change.

Maluf et al. (38) observed significant VAS reductions at the second evaluation, excluding sleep and mastication restrictions in both groups and ear symptoms in the GPR group. By the third evaluation, both groups exhibited significant improvements in TMJ pain and teeth clenching, while GPR showed significantly reduced cervicalgia. PPT increased in all muscles, indicating an improvement. By the third evaluation, SS showed a decrease, especially in the masseter, while GPR exhibited improvement only for the anterior temporalis. Electromyography resulted in a significant decrease at the second evaluation for the masseter, anterior temporalis, and sternocleidomastoid (SCM) muscles. By the third evaluation, differences remained significant only for SCM, with no significant differences between treatment groups (38).

In the von Piekartz and Ludtke (40) study, the OFG demonstrated significant improvements in the anamnestic questionnaire (AQ) between the second and third measurements, with significant differences between OFG and the UCG. Neck disability index (NDI) and AQ revealed significant distinctions, indicating a decrease in OFG compared to UCG. There were no differences in UCG at the second and third measurements, while OFG significantly differed from the initial assessment. Graded chronic pain status (GCPS) improved in OFG but not in UCG. PPT differences favored OFG. TMD signs showed significant differences, with a decrease in OFG. For the VAS, 85% of OFG patients improved in mouth opening, pain intensity, and NDI, with a significant difference in the second and third assessments compared to the first (40).

Of the five included studies, four reported significant improvements in headache-related outcomes following TMJ-related physiotherapy interventions. Garrigós-Pedrón et al. (37), von Piekartz and Ludtke (40), Calixtre et al. (36), and Maluf et al. (38) demonstrated significant reductions in headache intensity (via VAS, CAS), disability (HIT-6), or related pressure pain thresholds (PPT). Only Michelotti et al. (39) found no significant differences in headache outcomes between education and splint interventions. Overall, headache frequency and intensity improved more consistently in groups receiving multimodal physiotherapy that included TMJ and cervical approaches.

## 4 Discussion

The objective of our study was to systematically assess the existing literature regarding the effectiveness of physiotherapy in individuals with CH and TMD. Both Calixtre et al. (36) and Maluf et al. (38) exclusively enrolled female participants, which is consistent with the greater prevalence of headaches among females (1).

The treatment approaches differed across the five analyzed articles, and the results, except for one study (39), underscore the advantages of physiotherapy targeting the TMJ for CH. Three studies found that the experimental groups had better results for headache outcome measures (36, 37, 40). One study found better results for the control group (38). While the experimental group (GPR) did not yield significant results for headaches, the insights gained from the SS group contribute to the understanding that static stretching for the cervical spine, head, upper limbs, and mandibular muscles has a positive impact on headaches, which is related to our research question (38).

Two studies used a distinct diagnosis for headaches as defined by the ICDH-II and ICDH-III, which leads to chronic migraine and CGH (37, 40–42). The remaining three studies did not provide specific diagnoses for headache types (36, 38, 39). Examining the prevalence of headache types in TMD, primary headaches emerge as the most common. This observation leads to the hypothesis that many patients in the three articles predominantly present primary headaches (21). Therefore, the majority of the four studies encompass primary headaches (36–39), while one study incorporates CGH, which are secondary headaches (40). The divergence in headache types introduces discrepancies and may impact comparisons between results. In addition, four studies employed the RDC for TMD in diagnosing TMD (36, 38, 39), while one study utilized a diagnosis based on four signs. This approach facilitates a clear understanding and ensures homogeneity in patient types across all studies.

In this systematic review, two studies (37, 40) employed diverse treatments encompassing muscle interventions, cervical approaches, TMJ modalities, stretching, and home exercises. This approach aligns with various studies illustrating the positive outcomes of each intervention for TMD (43–46). It emphasized the effectiveness of integrating multiple interventions for treating headaches in patients with TMD. The investigation conducted by Maluf et al. (38) concentrated on stretching, and its findings are consistent with prior studies demonstrating the positive effects of cervical stretching in individuals with headaches and TMD (43, 44). The review of Fricton et al. (43) is a systematic review, with the study quality assessed, and is a relevant study. However, the study of Lee and Kim (44) is a single-center cohort study and lacks external validity and credibility in science (47). Results also aligned with a recent study showing the efficacy of mandibular stretching for TMD (48). Results are difficult to generalize as it is a pilot study with few participants (38, 49). Additionally, in opposition to research demonstrating the positive impact of GPR on diverse pathologies (50, 51), this study emphasized its lack of benefit for headaches (38). Discordance may be due to the evolution of GPR over the years.

Michelotti et al. (39) focused on education and splints, which did not impact headache outcomes. It contradicts previous studies concerning investigating education for TMD (45, 52), with both being systematic reviews showing good relevance. Additionally, incorporating home exercises (habit-reversal) in the education group did not influence headache outcomes. It contrasts sharply with the findings of Garrigós-Pedrón et al. (37) and von Piekartz and Ludtke (40). Literature also showed the positive impact of education and home exercises on TMD (45, 46). While the study of Shaffer et al. (45) is particularly pertinent, offering valuable insights, it is crucial to note that the investigation of Stuhr et al. (46) is a case study, posing challenges to the generalizability of its findings (53).

Calixtre et al. (36) focused their treatments on TMJ and muscles, similarly to Lee and Kim (44). The absence of detailed information regarding the specific home exercises performed in the studies creates ambiguity in reproducing their findings, possibly contributing to the divergence in results (37, 39, 40). Finally, there is also some divergence between the CG among the five studies, making it challenging to compare interventions. Two studies incorporate cervical treatments. The study of Garrigós-Pedrón et al. (37) includes treatment in the cervical region, explanation, manual therapy, techniques, tips, and home exercises. In comparison, von Piekartz and Ludtke (40) induce treatment at the craniocervical region, associated with techniques that were not specified. One study involves occlusal splints (39), while one SS (38). In analyzing these outcomes, it is also crucial to approach them cautiously, acknowledging the risk of bias identified within each study (Table 3).

## 5 Study strengths and limitations

Our review demonstrates multiple strengths, primarily as one of the few systematic reviews addressing TMD physiotherapy for individuals with CH. This systematic review provides an updated and focused analysis on the use of physiotherapy interventions specifically targeting the TMJ in patients with CH, an area that has received limited attention in previous reviews. While prior systematic reviews have generally explored broader physiotherapy interventions for TMD or various types of headaches, this review is among the first to evaluate TMJ-centered physiotherapy for CH using a strict PICOS framework and exclusively randomized controlled trials. All investigations integrated into this review strictly conform to the RCT design, enhancing the overall evidence level (54). We used the RoB2 tool to assess the quality of studies, which, according to Zeng et al. (55), is a robust assessment tool for assessing bias in RCTs. A comprehensive exploration of the limitations inherent in each study has been performed, and these limitations need to be considered to avoid overinterpretation of the results.

However, it is essential to acknowledge some limitations in our study. We find that TMJ physiotherapy confers advantages for individuals experiencing CH. However, the reliability of these findings is compromised by the varied quality of evidence in each study, as shown in the quality assessment in Table 3. Thus, many studies exhibited a high risk of bias, particularly in areas such as randomization, blinding, and missing outcome data. This high risk of bias may have led to the overinterpretation of outcomes, as flaws in study design could have resulted in inflated effect sizes. Therefore, caution must be exercised when interpreting the findings, as the true effects of TMJ physiotherapy may be different from what was reported. This diversity impacts the overall confidence in the study outcomes. Additionally, due to the small number of studies investigating different outcomes, with multiple methods of diagnosing headaches, a meta-analysis was not possible. The limited number of studies (*n* = 5) and the considerable heterogeneity in treatment protocols, outcome measures, and participant characteristics (e.g., types of TMJ physiotherapy interventions, headache classifications, and diagnostic methods) made it difficult to pool data for quantitative analysis. Variability in intervention protocols (e.g., TMJ physiotherapy, manual therapy, stretching) and outcome measures (e.g., headache frequency, intensity, quality of life) also contributed to the challenges in conducting a meta-analysis. When talking about the risk of bias, it is noteworthy that concealment and blinding were not consistently maintained across all studies assessed in our review. Studies showed that this can potentially influence the study outcomes (56, 57). In addition, in our review, studies did not involve exclusively physiotherapists. For instance, the investigation conducted by Michelotti et al. (39) enlisted a maxillofacial surgeon, and the study by Maluf et al. (38) did not specify the type of therapist involved. The primary objective of physical therapy is to alleviate pain, improve mobility, and strengthen weakened muscles, which is why physiotherapists are considered specialists in movements. Our systematic review focused on physiotherapy, which is why it creates discordance if clinicians other than physiotherapists performed treatment (38, 39). An inherent limitation that may arise from this is its potential to influence the obtained results (58). Finally, some of our studies failed to specify the precise nature of home exercises (37, 40), manual therapy techniques (37), coordination exercises, desensitization techniques, or neuromuscular treatments employed (40). None of the included studies specifically used biofeedback or similar integrative methods alongside TMJ physiotherapy. These observations reinforce the need for additional research in this domain.

Thus, while the review suggests promising outcomes from TMJ physiotherapy in managing chronic headaches and TMD, clinicians should consider the variability in treatment protocols and individual patient characteristics when applying these interventions in practice. Adapting treatments to the individual patient's needs, taking into account factors such as headache classification and TMD intensity, will enhance clinical results. Additionally, interdisciplinary collaboration between physiotherapists and other healthcare providers, including pain specialists and neurologists, is essential for creating comprehensive treatment plans for patients suffering from both TMD and chronic headaches.

Future research should aim to standardize intervention protocols and outcome measures, which would help reduce heterogeneity and facilitate more reliable comparisons. Once more studies with consistent methodologies and outcomes are available, a meta-analysis could be performed to better assess the efficacy of TMJ physiotherapy for chronic headaches. Larger studies with homogenous patient populations and more rigorous definitions of headache types will improve the generalizability and validity of the findings.

## 6 Conclusion

This systematic review explores the domain of physiotherapy targeted at the TMJ and its potential advantages for individuals suffering from TMD and CH. The comprehensive narrative synthesis of existing literature underscores the positive outcomes associated with physiotherapeutic interventions, shedding light on their potential efficacy in improving the symptoms and the overall well-being of patients affected. However, the review reveals a possible avenue for further exploration, particularly in understanding the details of various treatment techniques employed in physiotherapy. Notably, there is a need for more in-depth investigations concerning manual therapy, coordination exercises, home exercises, and neuromuscular interventions. A clear vision of these interventions is crucial to improve treatment protocols and patient outcomes. Interpreting the results requires a nuanced approach, and it is essential to pay attention due to the limitations discussed. In summary, while this systematic review highlights the positive role of physiotherapy in managing TMD and CH, it concurrently propels the research community toward future investigations involving different aspects previously stated.

## Data Availability

The original contributions presented in the study are included in the article/Supplementary Material, further inquiries can be directed to the corresponding author/s.
